# The Role of PPARs in Disease

**DOI:** 10.3390/cells9112367

**Published:** 2020-10-28

**Authors:** Nicole Wagner, Kay-Dietrich Wagner

**Affiliations:** Université Côte d’Azur, CNRS, INSERM, iBV, 06107 Nice, France; kwagner@unice.fr

**Keywords:** peroxisome proliferator-activated receptors (PPARs), toxicology, pharmacology, ligands, vascular, proliferation, cellular metabolism, adipogenesis, hypertrophic obesity, insulin-resistance, lipidomics, inflammation, kidney, cancer, tumor angiogenesis, peroxisome proliferator-activated receptor-γ coactivator-1α, non-alcoholic fatty liver disease (NAFLD), non-alcoholic steatohepatitis (NASH), fibrosis, Alzheimer’s disease (AD), addiction, alcohol, nicotine, opioids, psychostimulants, animal models, human studies

## Abstract

Peroxisome proliferator-activated receptors (PPARs) are nuclear receptors that function as ligand-activated transcription factors. They exist in three isoforms: PPARα, PPARβ/δ, and PPARγ. For all PPARs, lipids are endogenous ligands, linking them directly to metabolism. PPARs form heterodimers with retinoic X receptors, and upon ligand binding, they modulate the gene expression of downstream target genes, depending on the presence of co-repressors or co-activators. This results in a complex, cell type-specific regulation of proliferation, differentiation, and cell survival. PPARs are linked to metabolic disorders and are interesting pharmaceutical targets. PPARα and PPARγ agonists are already in clinical use for the treatment of hyperlipidemia and type 2 diabetes, respectively. More recently, PPARβ/δ activation came into focus as an interesting novel approach for the treatment of metabolic syndrome and associated cardiovascular diseases; however, this has been limited due to the highly controversial function of PPARβ/δ in cancer. This Special Issue of *Cells* brings together the most recent advances in understanding the various aspects of the action of PPARs, and it provides new insights into our understanding of PPARs, implying also the latest therapeutic perspectives for the utility of PPAR modulation in different disease settings.

Peroxisome proliferator-activated receptors (PPARs) were identified around three decades ago and are ligand-activated transcription factors of the nuclear hormone receptor superfamily comprising the following three subtypes: PPARα, PPARγ, and PPARβ/δ. In the current nomenclature system, PPARs are grouped as nuclear receptor 1C subfamily (PPARα–NR1C1; PPARβ/δ–NR1C2; PPARγ–NR1C3) [1]. PPARα regulates energy homeostasis, the activation of PPARγ causes insulin sensitization and enhances glucose metabolism, and the activation of PPARβ/δ enhances fatty acid metabolism [2]. Thus, the PPAR family of nuclear receptors plays a major regulatory role in energy homeostasis and metabolic function. The present Special Issue of *Cells* critically analyzes the protective and detrimental effect of PPAR modulation in dyslipidemia, adipocyte differentiation, cancer, kidney disease, cardiovascular disease, neurodegenerative disorders, addiction, non-alcoholic fatty liver disease, and steatohepatitis, and it provides a systematic evaluation of PPAR-mediated toxicology and applied pharmacology. 

The group of Valerio Costa describes in this Special Issue the generation of a model of human hypertrophic-like adipocytes, which is directly comparable to normal adipose cells and enables therefore evaluating the pathologic evolution toward a hypertrophic state as it is the case in hypertrophic obesity. Reduced neo-adipogenesis and dysfunctional lipid-overloaded adipocytes are hallmarks of hypertrophic obesity linked to insulin resistance. The authors performed a meticulous morphological and histological characterization of the immortalized hypertrophic-like adipocyte cell line. They further evidenced an unbalance of PPARγ isoforms in patients with hypertrophic obesity characterized by an increased relative amount of dominant negative and canonical transcripts (i.e., a higher PPARγ∆5/cPPARγ ratio). Their elegant model of hypertrophic-like adipocytes (HAs) mimics perfectly this increased PPARγ∆5/cPPARγ ratio as compared to mature adipocytes (MAs). Although cPPARγ expression was only slightly reduced in HAs (vs. MAs), PPARγ target genes (IRS2, ADIPOQ, FABP4, SLC2A4, PLIN1, PLIN2, and MRTFA) were highly reduced, explaining the impaired metabolic activity in hypertrophic adipose tissue. Only LPL was increased in HAs, which is in agreement with an increased expression also in subcutaneous adipose tissue from obese patients. The PPARγ-mediated induction of SLC2A4 (encoding the glucose transporter GLUT4) sets up an insulin sensitivity of adipose tissue, liver, and skeletal muscle. Costa’s group further tested for a correlation between the PPARγ∆5/cPPARγ ratio and SLC2A4 expression in vivo using patient samples from subcutaneous adipose tissues of obese individuals. SLC2A4 negatively correlated with the PPARGγ∆5/cPPARγ ratio in these patients. In conclusion, it is likely that the disequilibrium of PPARγ isoforms in the adipose tissue—especially the high PPARγ∆5/cPPARγ ratio—contributes to the insulin resistance frequently observed in patients with obesity. The unbalanced ratio between dominant negative and canonical isoforms in the adipose tissue favors the transcriptional repression of metabolic genes and impairs neo-adipogenesis, which are two main features of hypertrophic obesity and are correlated with insulin resistance and the onset of type 2 diabetes [3]. 

To improve the understanding of PPAR signaling in diverse vascular tissues, David Bishop-Bailey’s group investigated the in vivo generation of oxylipin PPAR ligands in different vascular tissues. Oxylipins are derived from the oxidation of polyunsaturated fatty acids (PUFAs), which act as important paracrine and autocrine signaling molecules. A subclass of oxylipins, the eicosanoids, has a broad range of physiological outcomes in inflammation, the immune response, cardiovascular homeostasis, and cell growth regulation. Using a targeted lipidomic analysis of ex vivo-generated oxylipins from porcine aorta, coronary artery, pulmonary artery, and perivascular adipose tissue, Bishop-Bailey’s group determined that cyclooxygenase (COX)-derived prostanoids were the most predominant oxylipin from all tissues. Interestingly, the coronary artery produced significantly higher levels of oxylipins from cytochrome P450 (CYP450) pathways than the other tissues investigated. The CYP450 pathway is to provide anti-inflammatory oxylipins that prevent processes of inflammatory vascular disease progression. The Toll-like receptor 4 ligand lipopolysaccharide (LPS) induced prostanoide formation in all the vascular tissues tested. Treatment of primary coronary artery smooth muscle cells (pCASMCs) with LPS induced a high expression of pro-inflammatory genes, which could be prevented by soluble epoxide hydrolase (sEH) inhibitor TPPU administration, demonstrating that endogenous CYP-derived epoxy–oxylipin PPAR ligands were strongly anti-inflammatory [4]. In experimental animal models, PPAR ligands reduced aortic atherosclerosis. In human patients, PPARα and PPARγ agonists showed some clinical efficacy in reducing cardiovascular events; in contrast, the PPARγ ligand rosiglitazone seemed to increase cardiovascular events (reviewed in [5,6]). There is interest in developing selective modulators and pan/dual-PPAR agonists, which might have increased efficacy and reduced side effects. Bishop-Bailey’s group presented work that suggests that endogenous oxylipin PPAR ligands are more likely to act as pan/dual or selective modulator-type agonists capable of reducing atherosclerosis. Importantly, the authors show that the CYP450 pathway in the coronary artery provides an anti-inflammatory tone, as the sEH inhibitor TPPU inhibited inflammatory mediators induced by TLR-4 activation in primary pCASMCs, thereby preventing processes of vascular disease progression. This important work describes for the first time the anti-inflammatory effects of sEH inhibitors in coronary tissue, supporting potential therapeutic cardioprotective actions of sEH inhibitors [4]. 

All PPARs are affecting angiogenesis. PPARα and PPARγ mediate mainly anti-angiogenic processes; in contrast, PPARβ/δ emerged as a pro-angiogenic factor (reviewed in [7]). 

The role of PPARβ/δ in cancer is controversial. Our group specifically wanted to address the impact of vascular PPARβ/δ for cancer growth and progression. We first tested the effects of the specific PPARβ/δ modulation on the proliferation of Lewis lung carcinoma cells (LLC1) in vitro. The PPARβ/δ agonist GW0742 decreased tumor cell proliferation, whereas the antagonist GSK3787 increased LLC1 proliferation in culture. Next, we treated LLC1 tumor-bearing mice with GW0742 and astonishingly observed increased cancer growth as well as enhanced metastases formation. Tumor sample analyses revealed an increased vascularization upon treatment with the PPARβ/δ agonist [8]. To further decipher the relevance of vascular PPARβ/δ for tumor progression, we used a mouse model with an inducible conditional vascular-specific overexpression of PPARβ/δ [9]. The inducible vascular-specific overexpression of PPARβ/δ promoted cancer angiogenesis, growth, and spontaneous metastases formation in vivo. To examine the transcriptome of gene expression patterns in PPARβ/δ overexpressing tumor-derived endothelial cells, we sorted endothelial cells from the LLC1 tumors of controls and mice with vascular PPARβ/δ overexpression and performed RNA sequencing. RNA sequencing of tumor-sorted endothelial cells revealed a high number of upregulated pro-angiogenic genes in response to PPARβ/δ increase. Combining top ten network analysis with a search for PPAR responsive elements divulged the platelet-derived growth factor (PDGF)/platelet-derived growth factor receptor (PDGFR) pathway, tyrosinkinase KIT (c-Kit), and the VEGF/vascular endothelial growth factor receptor (VEGFR) pathway as mediators of the pro-angiogenic tumor-promoting effect of PPARβ/δ. To determine the relevance for human pathophysiology, we also investigated human tumor samples and confirmed a high expression of PPARβ/δ in the tumor vasculature. The treatment of human umbilical vein endothelial cells (HUVEC) with the PPARβ/δ agonist GW0742 increased proliferation, which was accompanied by an upregulation of PDGFRB, PDGFB, and c-KIT expression. In contrast, the antagonist GSK3787 decreased the expression of these genes in HUVECs. In conclusion, we showed that PPARβ/δ favors tumor angiogenesis. Independently from their action on different cancer cell types, the therapeutic use of PPARβ/δ agonists appears to be dangerous [8]. Our group also stressed this point in the review “PPAR Beta/Delta and the Hallmarks of Cancer” for this Special Issue of *Cells.* Hanahan and Weinberg defined the interplay of cancer cell proliferation, angiogenesis, resisting cell death, evading growth suppressors, activating invasion and metastasis, enabling replicative immortality, deregulating cellular metabolism, and avoiding immune destruction as the didactic concept of the “hallmarks of cancer” to determine cancer growth and progression [10]. We outline in this review the effects of PPARβ/δ on these hallmarks and their underlying molecular mechanisms. In conclusion, we postulate that the therapeutic activation of PPARβ/δ results in alterations of the hallmark capabilities, favoring a pro-tumorigenic profile [11]. Given the initially proposed therapeutic potential of PPARβ/δ agonists as “exercise mimetics” and treatment for metabolic syndrome [12,13], nowadays, extreme caution should be applied when considering PPARβ/δ agonists for therapeutic purposes [11]. 

In the excellent review about PPAR ligands as candidates for the treatment of non-alcoholic fatty liver disease, Anne Fougerat and colleagues describe in a very detailed manner the factors contributing to the development, progression, and complications of liver steatosis, non-alcoholic fatty liver disease (NAFLD) and non-alcoholic steatohepatitis (NASH). PPARs are strongly implicated in glucose and lipid metabolism, and inflammation. The deregulation of these pathways is the underlying factor for the development of NAFLD and NASH. Therefore, PPARs have become attractive targets in the treatment of this disease complex. The review provides extensive information about experimental and clinical studies concerning the use of first generation and novel PPAR agonists in the treatment of NAFLD. Finally, perspectives for the development of safe PPAR agonists with improved efficacy targeting this pathology are discussed [14]. 

The review from the group around Jean-Louis Géant focuses on the crosstalk between Sirtuin 1 (Sirt1) and PPARs in metabolic diseases and the inherited disorders of the one-carbon metabolism. They describe the anti-oxidant and anti-inflammatory roles of Sirt1 and PPARs in metabolic diseases as well as the interplay of Sirt1 and PPAR activators in this setting. Sirt1 modulates the acetylation status of peroxisome proliferator-activated receptor-γ coactivator-1α (PGC-1α) and PPARγ. This leads to a certain level of protection against metabolic syndrome, as fatty acid oxidation, mitochondrial biogenesis, and white to brown adipose tissue differentiation are enhanced. A decreased expression and activity of Sirt1 is a common hallmark in a high-fat diet and also methyl donor deficiency (MDD). Notably in some inherited disorders of intracellular metabolism of vitamin B12, decreased Sirt1 expression plays an important role. In their review, the authors present promising data concerning the therapeutic use of Sirt1 agonists in inherited disorders of vitamin B12 metabolism [15].

Joseph M. Chambers and Rebecca A. Wingert focus in this Special Issue on the role of peroxisome proliferator-activated receptor gamma co-activator 1 alpha (PGC-1α) in kidney development and disease. While PGC-1α is required for proper renal development in zebrafish, the situation is not well studied in other species. In mice, PGC-1α deficiency seems to be compensated by PGC-1β. However, in different kidney cancer subtypes, the direct role of PGC-1α is variable; given that PGC-1α functions as a stress sensor of glucose depletion and enhances energy production, it might in general fuel cancer cell proliferation and disease progression. In contrast, in acute kidney injury and chronic and polycystic kidney disease, PGC-1α seems to be beneficial [16]. 

Nathalie Pierrots et al. describe in their contribution to this Special Issue the potential role of PPARα in the therapy of Alzheimers Disease (AD). Metabolic dysfunction (dyslipidemia, glucose metabolism impairment, and insulin resistance) is one risk factor leading to alterations of amyloid precursor protein (APP) processing and brain amyloid-β (Aβ) deposition, which are the main features of AD. PPARs are all expressed in the brain (PPARβ/δ exclusively in neurons and PPARα and γ in neurons and astrocytes). Among PPARs, PPARα is of special interest for the therapy of AD, as it is the only PPAR described to have neuronal functions implied in memory processes. Treatment with PPARγ agonists improved cognitive behavior in animal models of AD, while results from human trials were less successful. PPARβ/δ agonists decreased brain neuroinflammation, neurodegeneration, amyloid burden, and improved cognitive function in several animal models, and in a small human clinical cohort study, promising results on cognitive functions were reported. Many studies reported the beneficial effects of PPARα agonists on cognitive behavior in preclinical AD models. In conclusion, PPAR modulation might be a beneficial approach in AD therapy [17].

Justin Matheson and Bernhard Le Foll focused on the therapeutic potential of PPAR agonists in drug addictions. They provide a detailed overview of the key studies providing behavioral evidence for a role of PPAR agonists in modulating addiction-related behaviors in animal models as well as of the results from clinical and human laboratory studies of PPAR agonists in drug-related outcomes. While PPARβ/δ agonists do not seem to be implicated in the modification of addiction-related behaviors, both PPARα and PPARγ agonists are effective in reducing both the positive and negative-reinforcing properties of various drugs. Certain discrepancies between the animal and human study outcomes are explained in this review, and the potency and selectivity of PPAR ligands and sex-related variability in PPAR physiology discussed. Mainly, PPARα agonists seem to provide promising results against alcohol and nicotine addiction [18].

Finally, Yue Xi from the group of Zhiying Huang reviews the latest findings on PPAR-mediated toxicology and applied pharmacology. Due to their huge ligand-binding domain, PPARs are capable of binding a broad variety of compounds: endogenous and synthetic ligands as well as xenobiotics. Pharmacological ligands include full agonists, partial agonists, neutral antagonists, and inverse agonists. The information from 18 clinical trials using PPAR agonists for the treatment of diabetes and/or dyslipidemia is summarized. Furthermore, the authors discuss the positive impact of PPAR ligands in applied pharmacology, but they also detail the various toxicities observed by PPAR modulation, referring especially to the cardiovascular system, the liver, and the gastrointestinal system. They further highlight the beneficial and detrimental effects of PPAR interference in reproduction, development, cancer, muscle pathologies, kidney disease, and central nervous system pathologies. The authors conclude that there is still a substantial need to improve the comprehension of PPAR function in pharmacology and toxicology and their underlying molecular mechanisms, as only a few studies address an integrated network of relationships of all these aspects [19]. 

## Conclusions

Taken together, the Special Issue “The Role of PPARs in Disease” comprises the most recent studies that elucidate the physiological and pathological role of PPARs. The effects of PPAR modulation upon various pathological stimuli in cell/animal models of human diseases and in patients are discussed. The articles in this Special Issue further improve our understanding of the beneficial and detrimental consequences of PPAR modulation under physiological and pathological conditions, and they open new perspectives for the development of safer and more efficient PPAR-targeted therapies in the future. 
